# A Review of Magnetic Resonance (MR) Safety: The Essentials to Patient Safety

**DOI:** 10.7759/cureus.47345

**Published:** 2023-10-19

**Authors:** Aren Shah, Shima Aran

**Affiliations:** 1 School of Information, University of Michigan, Ann Arbor, USA; 2 Radiology/Breast Imaging, University of Texas Health Houston, Houston, USA

**Keywords:** safety training, mr risks, zoning, screening, mr safety

## Abstract

Nearly 40 million magnetic resonance imaging (MRI) scans are performed each year in the United States. MRI has become a relatively safe non-invasive diagnostic tool. To maintain a safe magnetic resonance (MR) environment, specific policies and safety procedures are required. The four zones of an MR site allow MR personnel to closely monitor and restrict the area. Screening patients with a questionnaire asking about implants, allergies to contrast agents, and other relevant medical information is important to safely perform an MRI scan. Providers may need to consider anesthesia for patients with claustrophobia who are unable to remain motionless. Radiologists and MR personnel need to be aware of some of the risks associated with MR and contrast agents. Safety training and knowledge of the emergency procedures in the MR environment are necessary to safely perform MR examinations.

## Introduction and background

Since the mid-1980s, magnetic resonance (MR) has become a useful diagnostic tool in clinical applications. MR is a non-invasive diagnostic tool and is relatively safe. Over the past several years, magnetic resonance imaging (MRI) has developed considerably. As a result, modern-day MRI uses stronger magnetic fields with high radiofrequency power. Policies and procedures are in place to ensure proper and safe use of MR. The static magnetic field and the time-varying gradient magnetic field lead to MR safety challenges [1]. Safety procedures need to be followed to protect patients, research subjects, and MRI personnel. Physicians, nurses, and other medical professionals who do not usually enter the MR suite need to be aware of MR safety procedures should they need to enter in urgent situations [1].

According to the American College of Radiology (ACR), there are three recommended positions that help ensure the proper use of MR: MR Medical Director for MR safety (MRMD), MR safety officer (MRSO), and MR safety expert (MRSE) [1]. The MRMD is a radiologist licensed to practice in that State. In the United States, the MRMD is liable for the safe execution of MR and oversees decisions regarding MR site access. To ensure the proper execution of MR, the MRMD establishes MR safety policies [2]. The MRSO, typically appointed by the MRMD, is responsible for following the MR practices established by the MRMD. A technologist typically fills the MRSO role and ensures safety procedures are followed [2]. The MRSE understands the physics behind MR safety issues and can guide to ensure the highest level of MR safety. The MRSE acts as an advisor to the MRMD and MRSOs [2]. These positions are important to ensuring a safe MR environment for patients, research subjects, and other MR personnel. This article aims to explore the policies and procedures necessary to maintain a safe MR environment and recognize ways to minimize risk in the MR environment.

## Review

MRI hardware

Main Magnet

MRI machines consist of three layers of magnets, which include a superconducting magnet, gradient coils, and body coils. The MR machine consists of a large coil of wire, which can only generate efficient magnetic fields in a narrow tube. The magnet in a 1.5 T scanner produces a magnetic field about 30,000 stronger than that produced by the Earth. MR machines consume a significant amount of energy and need to be maintained at a low temperature. Due to the strength of the magnetic fields, there are many safety considerations in place. The magnetic field can interfere with implants, so extensive screening is required [3]. The MR unit is divided into zones, which each have different restrictions. The main magnet is in Zone IV, which is the most restricted area of the MR unit. Most MR systems have the 5 Gauss field confined in the same room as the magnet, so that the fringe field does not affect other areas of the MR unit [3].

Gradient Systems

The gradient system encodes the MRI signal with the magnetic field gradient. The gradient systems have amplitudes of about 100 mT/m and slew rates of up to 200 mT/m/ms [3]. The coils change currents resulting in loud buzzing noises. The coils rapidly switch on and off to encode an MRI signal. These coils can cause peripheral nerve stimulation. Due to the loud noise, which can reach 100 dB or more, patients are given hearing protection [3].

Radiofrequency Coils

There are many different types of radiofrequency coils, including surface and volume coils. Radiofrequency coils are needed to excite nuclear magnetization to collect an MR signal. Surface coils are often used for spines, shoulders, and other smaller body parts. Surface coils need to be placed very close to the area being imaged to have a proper signal-to-noise ratio. Volume coils are typically used for whole-body imaging and larger regions of interest.

MRI magnets have superconducting coil windings, which are cooled by circulating liquid helium. The cooling is essential because even a small temperature rise can result in resistivity in coil windings and a reduced magnetic field. This process is known as quenching and can occur accidentally or in emergencies. During a quench, helium is quickly released from the MR machine using a quench pipe [1]. If the quench pipe fails, the helium can vent into the room, which can cause safety hazards. MR machines typically contain 1,500 to 2,000 L of cold liquid helium, which can cause the oxygen levels to decrease in the room [1]. Scan rooms should be equipped with an oxygen monitor as depletion of oxygen can result in a sudden increase in pressure. The quench pipes are designed to handle the enormous volume of helium gas, but there is a small chance the pipes could break [1]. Vent piping should be inspected to avoid issues should a quench be necessary. Quenching is a very rare event and can potentially damage the magnet. An emergency quench can be very costly and lead to downtime for the MRI machine.

MR environment: guidelines and regulations

Zoning and Screening

**Figure 1:**
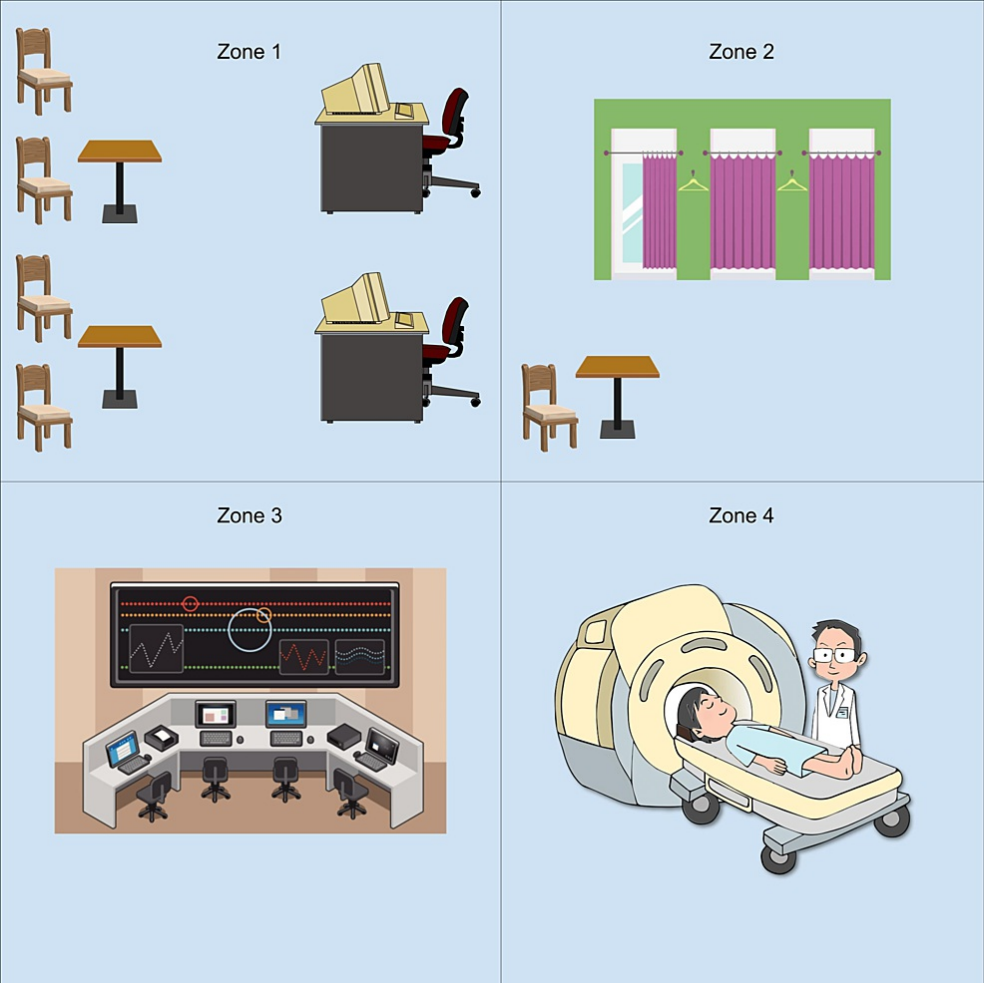
Four zones of the MR environment. These are the four zones of an MR unit. Zone I consists of the waiting area, which is outside the MR environment. Zone II is where patients change into MR-safe gowns and are screened for MR safety issues. Zone III consists of the control room, which is highly restricted. The scanner is in Zone IV, where there is the highest risk [1].

The MR site is divided into four zones, as illustrated in Figure 1, to mitigate the risks associated with MR. Each zone has different risks and is therefore monitored and restricted differently. Based on the ACR guidelines, which is the main manual used in the United States, the MR site is defined as below.

Zone I: This zone consists of public areas outside the MR environment, including waiting areas. These areas are unrestricted and where patients access the MR environment [3].

Zone II: This area is between Zone I and III. This zone is where patients are brought into their procedure [3]. Patients can be asked for their past medical history and be screened for MR safety issues in Zone II. Supervision is necessary for this area [1].

Zone III: This zone is highly restricted and is under close supervision by MR personnel. The scanner control room is in Zone III [3]. There can be potential danger from the interaction between unscreened people and the magnetic field of the scanner [1].

Zone IV: This area consists of the MR scanner magnet room, where there is the highest risk. This area must be highly supervised by trained personnel [3]. When unattended, the room door must be locked. If a medical emergency occurs, MR-trained personnel need to transport the patient to a magnetically safe location and begin basic life support if the patient’s condition allows [1].

MR personnel receive extensive training to ensure safety procedures are met. MR personnel working in Zones III and IV need to undergo training in MR safety and pass an MR safety screening process [1]. There are two levels of MR personnel. Level 1 MR personnel consists of individuals who pass the facility’s MR safety educational requirements. These requirements ensure that they understand MR safety and will not put themselves or others in danger [4]. Typically, Level 1 MR personnel consist of individuals who do not work in MRI on a full-time basis. Level 2 MRI personnel receive more extensive MR training with issues related to radiofrequency-related thermal loading and direct neuromuscular excitation [4]. They typically work full-time in MRI and are responsible for the safety of others in Zone IV [1]. Level 2 MR personnel need to monitor Level 1 personnel. The MRMDs identify individuals who are qualified to be Level 1 or Level 2 personnel [4]. Screening patients and research subjects before they enter the MR environment is important for their safety. The MR screening process identifies any foreign objects and medical implants. MR personnel may need to review other radiographs and reports of the implanted devices. The latest MR safety information regarding implants needs to be considered before the patient is placed in the MR environment [1]. When implants are close in proximity to neurovascular or soft tissue structures, the injury risk increases [3]. Implants made from non-ferromagnetic materials are usually labeled MR safe or MR conditional [4]. Ferromagnetic objects are usually labeled MR unsafe.

Ferromagnetic substances, which consist of iron, cobalt, and nickel, need to be restricted in Zone III. Ferromagnetic devices can be brought into Zone III under special circumstances. When they are in Zone III, they need to be secured and under proper supervision [1]. The ACR Committee on MR safety recommends using MR-safe gowns or scrubs in Zone IV, which can reduce the risk of burns from metallic fibers in clothing [1]. When ferromagnetic objects are brought into the MR environment, they can become dangerous projectiles and present a safety risk for the patient.

Implanted devices

**Figure 2:**
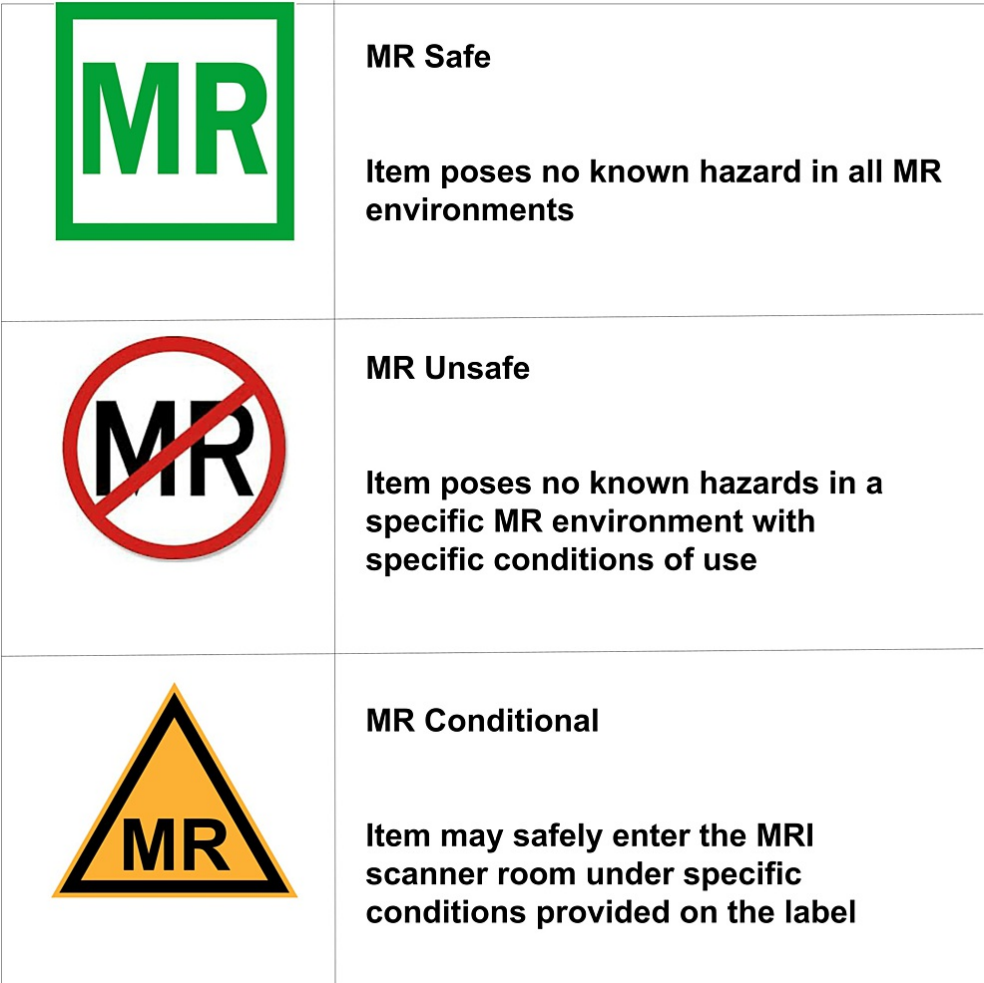
Three classifications for implanted devices.

Medical devices or implants are categorized as either MR safe, MR unsafe, or MR conditional. MR safe and unsafe designations can be determined by considering what a device is made of [5]. A screening checklist consists of screening for medical devices as any metallic device has the potential to cause harm within the MR unit [5]. MR staff can use a database that contains the safety ratings and recommendations for nearly all known medical devices at www.mrisafety.com [5]. When a device is labeled as MR conditional, the device is only compatible with certain conditions. To determine if the device is compatible, the magnetic field strength, magnetic field gradient, and the maximum specific absorption need to be considered [5]. Many medical implanted devices are either MR safe or unsafe. Several medical device manufacturers publish safety information and recommendations [5]. Reviewing these details is important to ensure a safe MR environment for the patient or research individual. Implanted medical devices may contain ferromagnetic material, which can interact with the magnetic field of the MR unit. Avoiding ferromagnetic objects in Zones III and IV can minimize risks. If the MR system operates at 1.5 T or less, the patient may be able to undergo MR when the implant contains no electrical power and is made from a non-ferromagnetic material [5]. Commonly used MRIs have a magnetic strength of 1.5-3 T. The magnetic field of the scanner can move an implant, which can present harm to a patient. The growth of tissue from medical devices can prevent the object from moving, which can minimize hazards in the MR environment. Waiting six to eight weeks is very important because, after that period, magnetic implants are less likely to be impacted by the magnetic field of the scanner. Whenever there is concern that the medical device may not remain in place, the individual should not be exposed to MR. A summary of the classifications of implanted devices is shown in Figure 2 [1].

Exposing a patient to an implanted medical device can sometimes lead to injury or death. Cardiac implantable electronic devices are affected by magnetic fields. While exposed to MR, the cardiac implantable devices can fail to pace or induce ventricular fibrillation, which can be fatal [1]. There are many other active implanted medical devices, including neurostimulators. Epicardial leads, which may contain ferromagnetic material, are often not removed by surgeons after a heart transplant. These leads need to be considered by MR personnel when evaluating risk.

MRI safety risks

Translational Force and Torque

Proper screening of the individual entering the MR unit is important to maintaining a safe MR environment. Magnetic objects in the MR unit are influenced by force and/or torque [5]. Due to the static or changing magnetic fields of the magnet, translational and rotational forces can interact with a magnetic object. The force can vary depending on the ferromagnetic composition, mass, and the gradient of the magnetic field strength [5]. Proper screening must account for magnetic objects as they can become dangerous projectiles in the MR unit.

Projectile Injury

Ferromagnetic objects brought into the MR unit can cause injury and equipment damage due to the strong translational and rotational forces [5]. Usually, these incidents involve objects external to the patient, including non-MR-imaging-compatible beds and chairs. While in the MR unit, these projectiles can cause serious injury and damage. Knowing the safety guidelines of all objects in the MR unit is important to maintaining a safe environment. The American Society for Testing and Materials (ASTM) develops the international consensus standards. ASTM F2503 provides definitions and labels devices as MR Safe, MR Condition, and MR Unsafe to avoid projectile injury. In addition to implant labels, ferromagnetic metal detectors are often used to avoid objects that can become unsafe [5].

Excessive Specific Absorption Rate

The specific absorption rate (SAR) is often used to define safe conditions for scanning patients with implants. SAR quantifies radiofrequency coil energy depiction in watts per kilogram [5]. To reduce the risks of thermal burns, Food and Drug Administration (FDA) guidance limits SAR whole-body exposure in patients with normal thermoregulatory function to 4.0 W/kg and 1.5 W/kg for all other cases [5]. SAR limits have also been declared for several MR-conditional devices to minimize thermal burn risk [5]. The SAR value significantly increases for individuals with implants because the body absorbs electromagnetic radiation at a higher rate. The increase in SAR value depends on multiple factors, such as the position, size, and orientation of the implant [6]. It is important to recognize that whole-body SAR measures the heating within the body and not within the implants [6]. Several factors, including the wavelength, radiofrequency transmit coil type, and position influence whether an implant will heat up in an MRI environment [6].

MRI units typically can provide an estimate of the SAR by using the total radiofrequency power transmitted per unit time with other patient data [5]. Ensuring that the SAR is within FDA limits is important to minimize the risk of thermal burns during the scan. There are multiple operating modes of scanner operation based on risk. The International Electrotechnical Commission (IEC) and the US FDA developed an international standard for MR safety. IEC 60601-2-33 recognizes three modes of scanner operation: normal operating mode, first-level controlled operating mode, and second-level controlled operating mode. Most MRIs are done under normal operating mode, and this mode is generally considered safe. MR scans that use the first controlled operating mode can cause physiologic stress. Therefore, the benefits and risks need to be assessed before performing this type of scan. Second-level control operating mode often presents significant risk and is usually only performed in research studies [6].

MR-Induced Burns

Potential thermal burns caused by radiofrequency fields can occur during an MRI. To reduce the risk of burns, it is important that patients wear appropriate clothing without metal components. Before the examination, the ferromagnetic detector system should be the last check to ensure that the patient does not have any metal components because even very small amounts of metal can cause serious burns [7]. In a previous case study, a patient wearing joggers experienced second-degree burn injuries due to the thin metal fibers in the vertical lines of joggers [7]. Eliminating metal in clothing is important to minimize the chances of thermal burns. In fact, more than 70% of MRI complications are related to thermal burns [7]. Therefore, detecting metal is imperative before the MRI examination begins. Certain tattoos have the potential to cause thermal injuries. Very dark tattoo pigment often contains iron oxide and other ferromagnetic metallic compounds [5]. These compounds can create an electric current, which can result in an increase in skin temperature leading to a burn [5]. Considering the pigment used for tattoos is important to minimize thermal burn risk in the MR environment.

MR During Pregnancy/Lactation

Based on guidelines from the ACR and the Society of Pediatric Radiology, MR exposure alone has not been shown to have a significant negative effect on the fetus [3]. Women during pregnancy can be exposed to non-contrast MR up to 3 T [1]. The recommendation is to avoid routine administration of gadolinium to pregnant patients. A decision to administer gadolinium to a pregnant patient should only be made when there is the potential for significant clinical benefit that outweighs the unknown risk of fetal exposure. This decision should be the product of a discussion between the radiologist, the patient, and the ordering physician [4]. Additionally, providers should evaluate potentially delaying the MR examination until after the pregnancy. MR personnel who are pregnant can continue to work but should stay outside of Zone IV during scanning [1].

Fetal MRI may be used to get more detailed images of a developing fetus and to identify fetal anomalies. If a possible problem is found on an ultrasound, a fetal MRI may be ordered. Current data does not suggest any harmful effects of MR on the developing fetus [4]. Some safety concerns regarding fetal MRI risk include acoustically related safety issues and heat deposition in tissues [4]. Studies on the safety of acoustic exposure during MRI on fetuses have not identified any adverse effects on the hearing of newborns [4]. Additionally, research involving MR exposure to pregnant pigs has mitigated safety concerns regarding heat deposition in tissues [4]. When conducting MRI at 3 T with standard SAR levels and a maximum scanning time of 30 minutes, no significant effects on the heating of fetal tissues have been demonstrated [4]. Ultimately, the patient’s provider must consider the clinical benefits and unclear risks of using MR to image a pregnant patient.

It is more common for patients after pregnancy to get an MRI scan. Studies evaluating excretion into breast milk reveal that less than 0.04% of the gadolinium dose reaches the milk [8]. Of the dose that reaches the milk, only 0.8% is absorbed by the infant. Because of the very small percentage of gadolinium excreted into breast milk, the available data suggest that it is safe to continue breastfeeding [4]. To reduce the amount of contrast in the mother’s milk, pumping and dumping with the continuation of nursing after six hours is generally recommended [8]. Ultimately, an informed decision to temporarily stop breastfeeding should be left up to the mother. If the mother remains concerned, she may abstain from breastfeeding from the time of contrast administration for a period of 12 to 24 hours [4].

MRI contrast agents

Gadolinium-based contrast agents (GBCAs) are regularly used in MRI to improve the clarity of a particular region being examined. There are safety concerns about the use of GBCAs for women during pregnancy [9]. As studies have reported inconsistent results, healthcare providers need to consider the benefits and risks as well as if there are other modalities, such as non-contrast MRI or ultrasound [9].

Gadolinium chelates are common MRI contrast agents but can have harmful side effects. Radiologists and MR personnel need to be aware of the effects of GBCAs. The frequency of adverse reactions to GBCAs is quite low. GBCAs pose a greater risk in patients with renal impairment [10]. For patients who need multiple GBCA-enhanced scans, new evidence may suggest an accumulation of gadolinium in the central nervous system [10]. There is a controversy regarding gadolinium retention in the body and the required FDA warning. Studies have confirmed gadolinium deposits in brain tissue, especially in the dentate nuclei and globus pallidus [11]. There is currently no data in humans or animals that show effects due to gadolinium deposition in the brain [11]. After a typical dose of 0.1 or 0.2 mmol/kg of gadolinium chelate, the frequency of acute adverse events ranges from 0.07% to 2.4% [3]. Potential effects include headache, dizziness, itching, nausea, and coldness at the injection site [3]. The frequency of very severe allergic reactions, including bronchospasm, rash hives, and urticaria, ranges from 0.004% to 0.7% [3]. The frequency of life-threatening reactions has frequencies ranging between 0.001% and 0.01% [3]. Severe reactions are rare, but radiologists and MR personnel need to have emergency procedures in place.

Supermagnetic iron-oxide nanoparticles (SPIONs) are an alternative potent MRI contrast agent. SPIONs have high magnetic susceptibility and provide negative contrast [10]. These particles exceed 10 nm in diameter and have an iron-oxide coating. The particle size is important when examining the SPION reflexivity. Larger particles that are at least 40 nm in diameter cannot be used for T1-weighted imaging [10]. T1 reflexivity is greater at 1.5 T for smaller SPIONs [10]. Ferumoxytol is the only SPION formulation approved by the US FDA. Ferumoxytol is sometimes used for pediatric MRI because of the long circulatory half-life of about 12-14 hours [10]. These particles provide strong vessel-to-tissue contrast. There are safety considerations when using ferumoxytol. Adverse drug reactions from ferumoxytol are more common compared to using GBCAs [10]. Using ferumoxytol has caused serious allergy reactions [10].

Another alternative contrast media is manganese-based contrast agents. Similar to gadolinium, manganese can induce magnetic relaxation via a similar mechanism [10]. The FDA approved a manganese-based contrast agent known as mangafodipir, but it was discontinued commercially. Mangafodipir partially dissociates in the bloodstream and is taken up by hepatocytes, which makes it suitable for hepatobiliary imaging [10]. Manganese is considered relatively safe because it is essential for life and the body has mechanisms to control manganese levels [10]. Therefore, manganese could have lower toxicity compared to gadolinium. Despite the benefits of manganese-based contrast agents, challenges have limited its widespread use. Manganese-based contrast agents have lower thermodynamic stability and kinetic inertness compared to GBCAs [10]. Additionally, manganese-based contrast agents may provide more limited information. However, recent reports of high-relativity and stable manganese-chelate molecules support the future possibility of increased use of magnesium in contrast media [10].

Pediatric MRI

MRI is often used for pediatric patients because it is generally considered safe and eliminates the risk of radiation. The MR safety guidelines still apply, but family members can accompany pediatric patients. Therefore, they need to undergo proper screening. In Zones III and IV, Level 2 MR physicians often limit one accompanying individual [4]. Pediatric patients should be asked the necessary precautionary questions twice to ensure a safe MR scan. The pediatric patient should be asked the necessary safety questions once their parents are present so MR personnel are aware of any potential risks [4]. Some pediatric patients are given general anesthesia so that quality MR scans can be obtained. Due to the small-bore sizes, some patients, including children, may experience claustrophobia during the examination. To help reduce claustrophobia, providers should speak to their patients about the MRI examination. To reduce claustrophobia, larger bore sizes and open MRI systems can be used [3]. Claustrophobic patients who are unable to remain still may need to consider anesthesia during the examination. As MRI scans are sensitive to motion and take time, some patients may be unable to remain motionless. Providers should inform their patients about the risks and benefits of sedation [3].

MRI is often associated with long acquisition times, which is a downside of using MRI for pediatric patients [12]. Sedation is often necessary to obtain quality images. Several techniques have been developed to lower the acquisition time, which can decrease the need for sedation and improve image quality. Some of these techniques include parallel imaging, single-shot acquisition, and controlled aliasing techniques [11]. Artificial intelligence can perform image reconstruction by generating images based on pre-established assumptions. Image reconstruction technology has become commercially available and approved by the US FDA [12].

Safety training and emergency procedures in an MR environment

MR personnel need to be aware of the potential adverse effects of MRI. MRI safety training that includes the technical and medical background of MRI should be completed annually. To understand the dangers associated with MR, training programs should include hands-on demonstrations of the effects of ferromagnetic objects [13]. Another important aspect of the training program is screening patients to learn about any ferromagnetic objects, implants, allergies to contrast agents, kidney disease, and other past relevant medical information [13]. Knowing this information is imperative to avoiding injury. As the majority of MR complications are related to burns, an MRI safety course should discuss ways to minimize the chances of thermal burns [14]. The most effective way to reduce the risk of burns is to require patients to change into MR-safe clothing. Some clothing may contain small amounts of metallic fibers, which are often listed on the label of a particular piece of clothing [14]. Additionally, MR personnel should understand the hearing risks associated with MR. During the scan, there can be very high acoustic noise levels in the gradient system [15]. Patients need to wear earplugs and headphones to avoid hearing damage [15]. As severe allergic reactions to gadolinium chelates occur, MR personnel need to be trained on how to address these emergencies.

## Conclusions

MR is a safe, non-invasive, and very useful diagnostic tool for several clinical applications. Understanding the safety policies and procedures is necessary to ensure the safe use of MR for patients and research subjects. Dividing the MR site into four zones, with each restricted differently, is important for proper organization. Additionally, proper screening is necessary to evaluate a patient’s risk in the MR unit. Avoiding ferromagnetic objects is important to avoiding injury and damage to the MR equipment. MR staff need to be aware of MRI safety risks, which include projectile injury, excessive SAR, thermal burns, and side effects of GBCAs. Proper organization and screening in the MR unit as well as detailed training of MR personnel are important steps to ensure safe use of MR.
